# Rickettsial *ompB* Promoter Regulated Expression of GFP_uv_ in Transformed *Rickettsia montanensis*

**DOI:** 10.1371/journal.pone.0008965

**Published:** 2010-01-29

**Authors:** Gerald D. Baldridge, Nicole Y. Burkhardt, Adela S. Oliva, Timothy J. Kurtti, Ulrike G. Munderloh

**Affiliations:** Department of Entomology, University of Minnesota, St. Paul, Minnesota, United States of America; Charité-Universitätsmedizin Berlin, Germany

## Abstract

**Background:**

*Rickettsia* spp. (Rickettsiales: Rickettsiaceae) are Gram-negative, obligate intracellular, α-proteobacteria that have historically been associated with blood-feeding arthropods. Certain species cause typhus and spotted fevers in humans, but others are of uncertain pathogenicity or may be strict arthropod endosymbionts. Genetic manipulation of rickettsiae should facilitate a better understanding of their interactions with hosts.

**Methodology/Principal Findings:**

We transformed a species never associated with human disease, *Rickettsia montanensis*, by electroporation with a TN5 transposon (pMOD700) containing green fluorescent protein (GFPuv) and chloramphenicol acetyltransferase (CAT) genes under regulation of promoters cloned from the *Rickettsia rickettsii ompB* gene, and isolated a Chloramphenicol-resistant GFP-fluorescent rickettsiae population (Rmontanensis700). The Rmontanensis700 rickettsiae contained a single transposon integrated near an acetyl-CoA acetyltransferase gene in the rickettsial chromosome. Northern blots showed that GFPuv and CAT mRNAs were both expressed as two transcripts of larger and smaller than predicted length. Western immunoblots showed that Rmontanensis700 and *E. coli* transformed with a plasmid containing the pMOD700 transposon both expressed GFPuv proteins of the predicted molecular weight.

**Conclusions/Significance:**

Long-standing barriers to transformation of rickettsiae have been overcome by development of transposon-based rickettsial transformation vectors. The *ompB* promoter may be the most problematic of the four promoters so far employed in those vectors.

## Introduction

The genus *Rickettsia* (Rickettsiales: Rickettsiaceae) consists of small, obligately intracellular Gram-negative bacteria that have been studied principally as established and newly emerging human pathogens transmitted by specific blood-feeding arthropods [1], [2]. That historical view of rickettsiae as essentially a group of vector-borne pathogens of humans has yielded to a new appreciation of the genus as a group of bacterial endosymbionts that are widely distributed in a diverse range of arthropods and other invertebrates and even protozoa and plants [3], [4]. Nevertheless, the majority of known *Rickettsia* spp. are associated with arthropods and in some cases function as reproductive manipulators of their hosts [5], [6]. The close and apparently ancient relationship of the genus *Rickettsia* with arthropods is consistent with the probable evolution of vertebrate-pathogenic rickettsiae from nonpathogenic ancestors [4], [7].

Rickettsiae that are widely distributed in North American ticks that readily feed on humans but are not associated with human disease include the recently isolated (TJ Kurtti, unpublished) *Ixodes scapularis* endosymbiont [8], [9], [10], *Rickettsia peacockii and R. montanensis*. *R. peacockii* was isolated from *Dermacentor andersoni* collected in Montana [11] and Colorado [12], occurs at high prevalence in *D. andersoni* and *Dermacentor variabilis* tick populations in the northwestern USA and western Canada [13], [14]. *R. peacockii* is not pathogenic in laboratory animals and may be a limiting factor in the incidence of Rocky Mountain Spotted Fever (RMSF) by interfering with transovarial transmission of *R. rickettsii* in *Dermacentor* ticks [15], [16]. *R. montanensis* was isolated from *D. andersoni* and *D. variabilis* ticks collected in Montana [17], was originally described as *R. montana* [18], and is widely distributed in *Dermacentor* tick populations in the northern and eastern USA [17], [19], [20], [21], [22], [23], [24]. *R. montanensis* is not pathogenic in laboratory animals [25], [26], [27], [28]. Similar to *R. peacockii*, *R. montanensis* may affect the epidemiology of Rocky Mountain spotted fever by interference with transovarial transmission of *R. rickettsii* infections in ticks [15], [16], [29], and by conference of protective immunity to infected mammalian hosts subsequently exposed to *R. rickettsii* [25], [26]. Recent studies have begun to explore the dynamics of the *D. variabilis* gene expression response to infection with *R. montanensis* [30], [31], [32], which is perhaps the best established laboratory model for study of the interactions of a *Dermacentor* tick host with a spotted fever group *Rickettsia* spp. that is not a human pathogen.

The study and comparative analysis of nonpathogenic *Rickettsia* spp. with their pathogenic congeners may illuminate the mechanisms of rickettsial pathogenesis. The nonpathogenic *Rickettsia* spp. are attractive targets for development of transformation technologies that can facilitate those studies and be subsequently extended to transformation of pathogenic rickettsiae. Until recently, transformation of rickettsiae was frustrated by lack of appropriate selectable markers and a reliable transformation system [33]. Those barriers are now yielding to change in recommended drug treatments for rickettsiosis patients [34], [35], [36] and development of TN5 and Himar1 transposon vectors for transformation of rickettsiae. Transformation with a TN5 transposon carrying a rifampicin resistance factor gene under regulation of the *Rickettsia prowazekii rpsL* promoter allowed isolation of gene knockout mutants of *R. prowazekii* [37], [38]. TN5 transposomes were also used to transform *Rickettsia monacencis* to express chloramphenicol acetyltransferase resistance factor (CAT) and green fluorescent protein (GFPuv) genes under regulation of *ompA* promoters cloned from *Rickettsia rickettsii* [39], [40]. Northern blot and RT-PCR analyses demonstrated expression of mRNA transcripts of the expected lengths but TN5-mediated transformation of *R. monacensis* with a GC-rich gene encoding the DsRed2 fluorescent reporter under regulation of the powerful *R. rickettsii ompB* promoter resulted in expression of two unexpected mRNA species that were not translated [39]. *R. prowazekii* was subsequently transformed with a Himar1 transposon that carried rifampin resistance and GFPuv expression cassettes derived from the above transposon vectors as well as a Himar1 transposase gene under regulation of the *Borrelia flg* promoter [41].

The successful transposon-based transformations of typhus group (*R. prowazekii*) and spotted fever group (*R. monacensis*) rickettsiae implied general applicability to other rickettsiae. To validate that concept and to further evaluate the utility of the *ompB* promoter, we chose *R. montanensis* as a target for transformation with a TN5 transposon carrying genes encoding the CAT and GFPuv markers under regulation of independent *ompB* promoters. We obtained Chloramphenicol-resistant *R. montanensis* transformants that expressed GFPuv from a single chromosomally integrated transposon, but they were substantially less fluorescent than the *R. monacensis* GFPuv transformant. Northern blot analyses indicated that both genes were expressed as two transcripts of unpredicted length as in the case of *ompB* promoter regulated expression of DsRed2 transcripts in *R. monacensis*. However, Western blot analyses demonstrated expression of GFPuv protein of the predicted molecular mass of 26 kDa in contrast to lack of DsRed2 protein expression in *R. monacensis*. The results confirmed utility of the recently developed transposon-based rickettsial transformation technologies, but indicated the need for careful evaluation of the existing and candidate promoters that may be utilized in further development of rickettsial transformation vectors. The Rmontanensis700 transformant can be used to enhance the *R. montanensis* in *D. variabilis* model system through visualization of GFP-fluorescent rickettsiae in unfixed tick tissues.

## Materials and Methods

### Growth, Purification and Transformation of Rickettsiae

*Rickettsia montanensis* isolate M5/6 [17] and *Rickettsia monacensis* isolate IrR/Munich [42] were grown in the *I. scapularis* ISE6 cell line [43] maintained in L-15B300 medium as described previously [43]. Rickettsiae were released from infected host cells suspended in L-15B300 medium by aspiration (5 times) through a bent 27 gauge hypodermic needle attached to a 5 ml syringe. The host cell lysates were passed through a 2.7 µm pore size syringe filter to remove cellular debris, and the filtrates were centrifuged at 15,000×*g* at 4°C for 5 min to collect rickettsiae. The rickettsiae were resuspended and transformed by electroporation with the pMOD700 transposon or used to prepare genomic DNA as described previously [39]. Transformant *R. montanensis* were isolated and maintained under Chloramphenicol (CHL) (Roche, Indianapolis, IL) as described previously [39].

### The pMOD700 Transposon Vector

The pMOD700 transposon vector was constructed with the same materials and methods used in construction of the closely related pMOD658 and pMOD601 rickettsial transformation vectors as described previously [39]. All enzymes and other reagents were from Invitrogen (Rockville, MD) or Promega (Madison, WI) and were used according to manufacturers' recommendations. In brief, expression cassettes containing the CAT and GFPuv genes under regulation of the *R. rickettsii* isolate Hlp#2 *ompB* gene promoter and *ompA* gene transcriptional terminator were assembled and inserted into the pMOD-2<MCS> transposon construction vector (Epicentre, Madison, WI) to obtain the pMODompBCAT\GFPuv700 (pMOD700) rickettsial transformation vector. Transposons were released from the vector and used to prepare transposomes for electroporation of rickettsiae according to the manufacturer's suggested protocol.

### Rickettsial Genomic DNA Preparation and Southern Blot Analyses

Rickettsial genomic DNA was prepared from resuspended rickettsiae as described previously [39]. For Southern blots, 100 ng rickettsial DNA was digested overnight with Hind III, MunI or PstI at 37°C, electrophoresed on1% agarose/TAE gels, transferred onto positively charged nylon membrane and hybridized with digoxigenin-labelled CAT and GFPuv gene probes as described previously [39].

### Characterization of the Transposon Integration Site

The pMOD700 transposon integrated into the *R. montanensis* genome was recovered by plasmid rescue cloning of the CAT gene. XhoI-digested and EcoR1-digested DNA from transformant *R. montanensis* cells was treated with the DNA Terminator^R^ End Repair Kit, ligated into the pSMART-LCKan vector with Clone Smart DNA ligase and electroporated into *E. cloni*™ 10 G electrocompetent cells according to the manufacturer's suggested protocols (Lucigen, Madison, WI). Electroporated cells were plated on Luria-Bertani agarose plates containing 50 µg per ml of CHL. Resistant colonies growing at 37°C were cultured in LB medium with 50 µg per ml of CHL. LCKan plasmid DNA bearing the rescued pMOD700 transposon was prepared from resistant cultures with the High Pure Plasmid Isolation Kit (Roche). Rickettsial DNA flanking the pMOD700 transposon was sequenced (ABI 377 automated sequencer - Advanced Genetic Analysis Center, University of Minnesota) using the pMOD SqRP and SqFP primers and the LCKan vector SL1 and SR1 primers.

### RT-PCR and Northern Blot Detection of CAT and GFPuv mRNAs

RNA was prepared from bacteria with the RiboPure Kit (Ambion, Austin, TX) according to the manufacturer's protocol, including Dnase I treatment. Fifty ng aliquots of RNA were used as template in Access RT-PCR System (Promega) reactions according to the manufacturer's protocol with CAT and GFPuv gene primers as described previously [39]. A second round RT-PCR reaction was executed with a nested GFPuv gene primer pair: TTCTGTCAGTGGAGAGGGTGAAGGTGATGC and CCATTCTTTTGTTTGTCTGCCGTG. The reaction products were electrophoresed in 1% agarose/TAE gels and stained with EtBr or SYBR Green (Molecular Probes, Eugene, OR) for UV visualization. For Northern blots, RNA was prepared from bacteria stored in RNAprotect Bacteria Reagent and recovered from RNeasy kit spin columns according to the manufacturer's protocols (Qiagen, Valencia, CA). RNA was electrophoresed on 1% agarose/formaldehyde gels in 1x MOPS running buffer, transferred onto positively charged nylon membrane and hybridized with digoxigenin-labelled CAT and GFPuv probes as described previously [39].

### Western Immunoblot Analysis of GFPuv Expression

Expression of GFPuv protein was analyzed by Western immunoblotting. Bacteria were boiled in 2 volumes of Tris buffer-2%SDS-660 mM β-mercaptoethanol-bromophenol blue tracking dye. Protein concentration in the extracts was determined with a Bradford Protein Assay kit (Bio-Rad, Hercules, CA). Proteins (10 µg) were separated by electrophoresis in a GeneMate Express mini-SDS-polyacrylamide gradient gel (8 to 16%; ISC BioExpress, Kaysville, UT) and blotted onto Immobilon-P membranes (Millipore, Bedford, MA). GFP-specific bands were detected with goat anti-GFP IgG (Rockland Immunochemicals, Gilbersville, PA) as described previously [39].

### Visualization of GFP-Fluorescent Rickettsiae

Tick ISE6 cells or DVE1 cells derived from *D. variabilis* [44] were infected with transformant *R. montanensis* and spun onto microscope slides using a Cytospin centrifuge (Shandon Southern Instruments, Sewickly, PA). The GFP-fluorescent rickettsiae were visualized on a Nikon C1 Imaging Confocal Microscope equipped with a GFP filter and digital imaging system at the University of Minnesota, College of Biological Sciences, Imaging Center.

## Results

### Structure of the pMOD700 Rickettsial Transformation Vector

**Figure 1:**
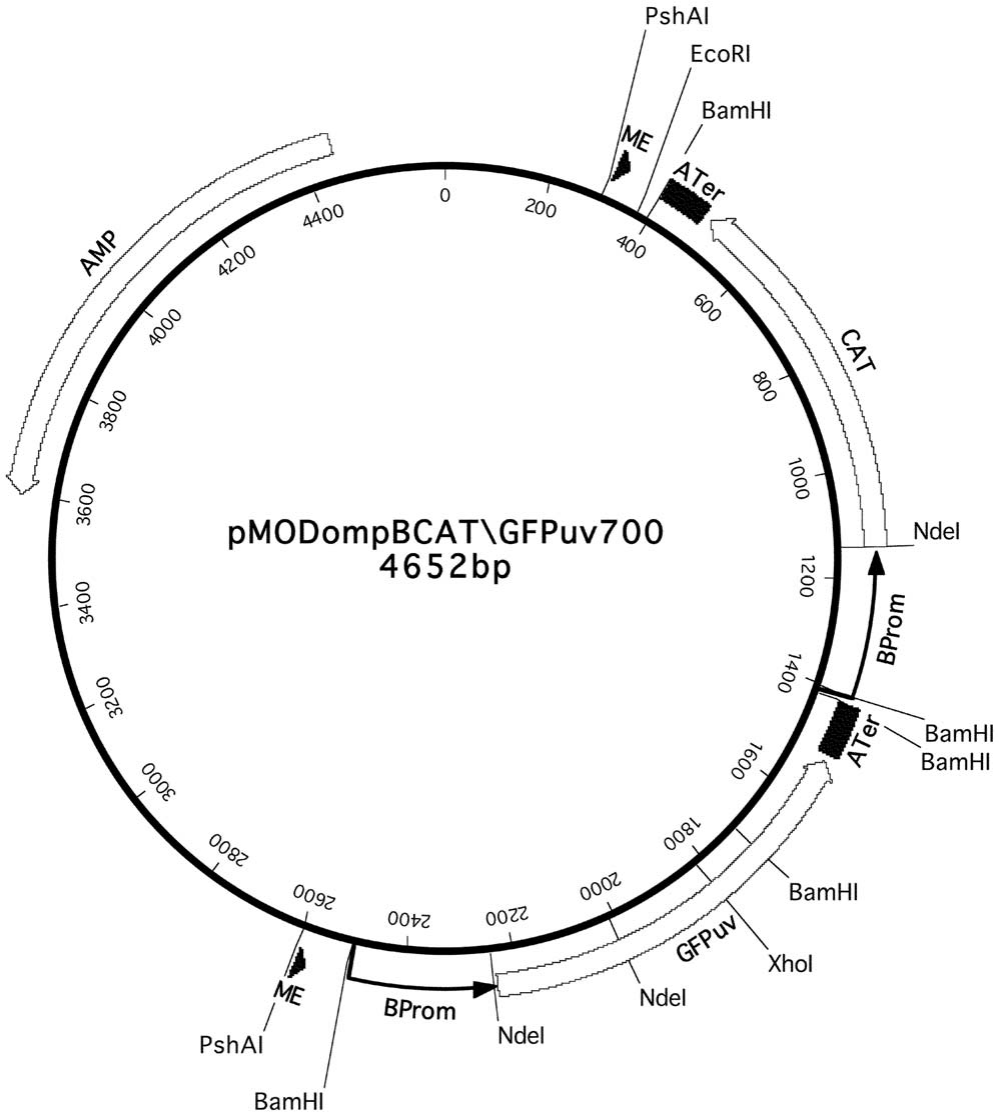
The pMODompBCAT\GFPuv700 transformation vector. Relative positions of the following functional elements are indicated: the 19 nucleotide mosaic element (ME) sites recognized by EZ::TN Transposase; the rickettsial *ompB* promoters (BProm) and *ompA* transcription terminators (ATer); the green fluorescent protein (GFPuv), chloramphenicol acetyltransferase (CAT) and ampicillin resistance (AMP) genes with direction of transcription indicated by arrow heads. Lines indicate restriction enzyme sites involved in Southern blot experiments, plasmid rescue cloning, and excision of the transposon from the vector for incubation with EZ::TN transposase to form transposomes for electroporation.

The pMODompBCAT\GFPuv700 (pMOD700) vector was constructed from the same PCR-amplified *R. rickettsii ompB* promoter and *ompA* terminator transcriptional regulatory elements, and the GFPuv and CAT genes that were used to construct the pMOD658 and pMOD601 rickettsial transformation vectors [39]. The pMOD700 vector, depicted schematically in Fig. 1, has independent GFPuv and CAT gene expression cassettes, each with an *ompB* promoter and *ompA* terminator, arranged in tandem head to tail orientation within the transposon. The overall structure of the pMOD700 vector therefore resembled that of the pMOD658 vector in which the genes were under regulation of *ompA* promoters and terminators [39]. In the pMOD601 vector, a DsRed2 reporter gene under regulation of the *ompB* promoter was placed in opposite head to tail orientation to the CAT gene under regulation of the *ompA* promoter, with a single *ompA* terminator located between the 3′ ends of the CAT and DsRed2 genes [39].

### Isolation and Visualization of Transformant *R. montanensis*

Rickettsiae, electroporated with pMOD700 transposomes and inoculated onto ISE6 cells, were selected with CHL at 2 *u*g per ml with biweekly medium changes. Extracellular rickettsiae were no longer visible after several days but by 3 months after electroporation, several small, discrete plaques were noted in the cell layer. At that time, CHL was temporarily withdrawn, and two days later, the culture was fully infected. Control rickettsiae did not survive CHL selection. The CHL-resistant rickettsiae were designated as *R. montanensis* pMODompBCAT\GFPuv line 700 (Rmontanensis700) and were serially passed by inoculation of 0.1 ml of infected culture onto new ISE6 cell cultures maintained with 1 *u*g of CHL per ml and are currently at the 29^th^ passage.

**Figure 2:**
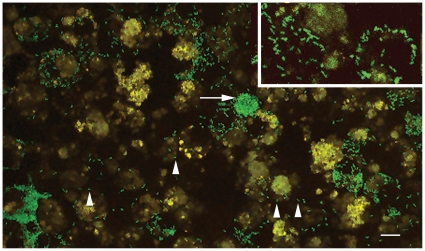
GFPuv-fluorescent *R. montanensis* transformant cells in tick DVE1 cells. Arrow indicates dense cluster of fluorescent rickettsiae and arrowheads indicate dispersed single (left) and diplococcal (right) rickettsiae. Scale bar indicates 10 µm. Inset at upper right approximately 2.5x zoom.

Although the Rmontanensis700 rickettsiae were resistant to CHL, GFPuv-fluorescent rickettsiae were difficult to visualize on a Nikon Eclipse E400 microscope equipped with epifluorescent illumination and a fluorescein isothiocyanate filter. In contrast, the GFPuv-fluorescent Rmona658 transformant rickettsiae [39] were easily visualized on the same microscope. However, the Rmontanensis700 rickettsiae were clearly GFPuv-fluorescent when examined on a confocal microscope equipped with a more sensitive digital camera. Fluorescent rickettsiae were visible in ISE6 cells and in DVE1 cells (Fig. 2) as widely dispersed single and diplococcal bacteria (arrowheads) and as dense clusters (arrow) that increased in size as the infection progressed.

### Determination of the pMOD700 Transposon Integration Site in the *R. montanensis* Chromosome

**Figure 3:**
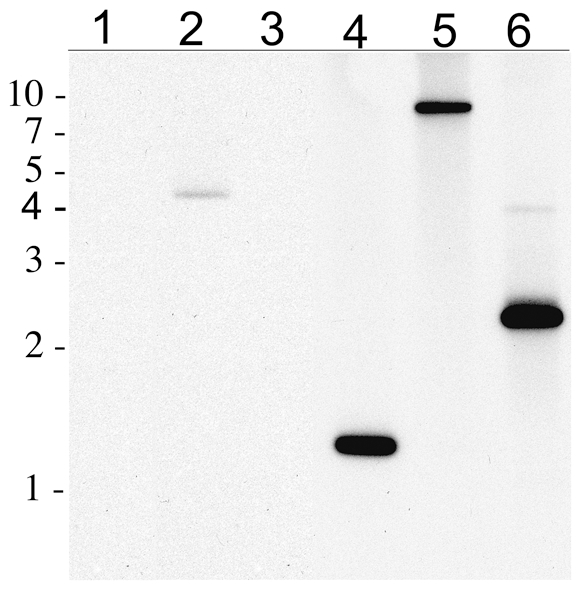
Southern blot detection of the pMOD700 transposon CAT gene in DNA of the Rmontanensis700 transformant. Lane 1: contained a 1 kbp-DNA marker ladder with relative migration positions indicated at left. Lane 2: 10 picograms of pMOD700 plasmid digested with XhoI. Lane 3: 100 nanograms of untransformed *R. montanensis* genomic DNA digested with HindIII. Lanes 4, 5 and 6 contained 100 nanograms of transformant Rmontanensis700 DNA digested with HindIII, PstI and MunI, respectively. The blot was hybridized with a CAT gene probe.

We confirmed integration of the pMOD700 transposon in the *R. montanensis* chromosome by Southern blot analysis. Rickettsial DNA extracts were electrophoresed in a 1% agarose gel and transferred onto a nylon membrane. The pMOD700 plasmid digested with XhoI, which cleaves once within the GFPuv gene (Fig. 1), hybridized with a CAT gene probe as the expected single band migrating at approximately 4.6 kbp (Fig. 3, lane 2) relative to DNA size markers (lane 1). Untransformed *R. montanensis* DNA digested with HindIII, which cleaves near the 3′ end of the *ompB* promoters in the pMOD700 transposon (Fig. 1), did not hybridize with the CAT probe (Fig. 3, lane 3). Rmontanensis700 DNA digested with HindIII hybridized to the CAT probe as a single band migrating at approximately 1.3 kbp (lane 4), consistent with integration of a single pMOD700 transposon approximately 0.5 kbp from a HindIII site in the rickettsial chromosome. Integration of a single transposon was confirmed by hybridization of Rmontanensis700 DNA digested with PstI and MunI, which respectively cleave once near the 5′ end of the transposon and within the GFPuv gene (Fig. 1). We observed a single PstI-digested band that migrated at approximately 9 kbp (lane 5) and a predominant MunI-digested band at approximately 2.3 kbp as well as a very faint larger band probably due to incomplete digestion (lane 6), consistent with integration of the transposon approximately 1 kbp from an MunI site in the rickettsial chromosome.

We identified the pMOD700 transposon insertion site by sequencing the CHL marker-rescued transposon and flanking *R. montanensis* DNA. All rescued clones contained a single copy of the transposon with intact mosaic element sequences at each end that were flanked by the 9-bp insertion site sequence duplications typical of EZ::TN transposase. All clones contained the same 9-bp duplication (ATTTTATGG), indicating that the Rmontanensis700 transformant population was derived from a single transposon integration event. The sequence data revealed the presence of HindIII and MunI sites located approximately 0.5 and 1 kbp, respectively from the CAT terminal end of the integrated transposon, consistent with the Southern blot data (see above).

BLAST alignment of the *R. montanensis* sequences flanking the integrated transposon to the *R. conorii* genome (Genbank accession no. AE006914) showed that the transposon had integrated within a sequence that was highly similar to that of a *R. conorii* gene (RC1133) encoding a predicted protein of unknown function. The 9 bp insertion site duplication at the transposon ends corresponded to nucleotide positions 1,048,051–1,048,059 in the *R. conorii* genome. The nearby *R. conorii* split gene (RC1134 and RC1135) that encoded an acetyl-CoA acetyltransferase was also present on the rescued *R. montanensis* sequence as an approximately 96% similar split gene.

### GFPuv and CAT mRNA Expression in Transformant *R. montanensis*

**Figure 4:**
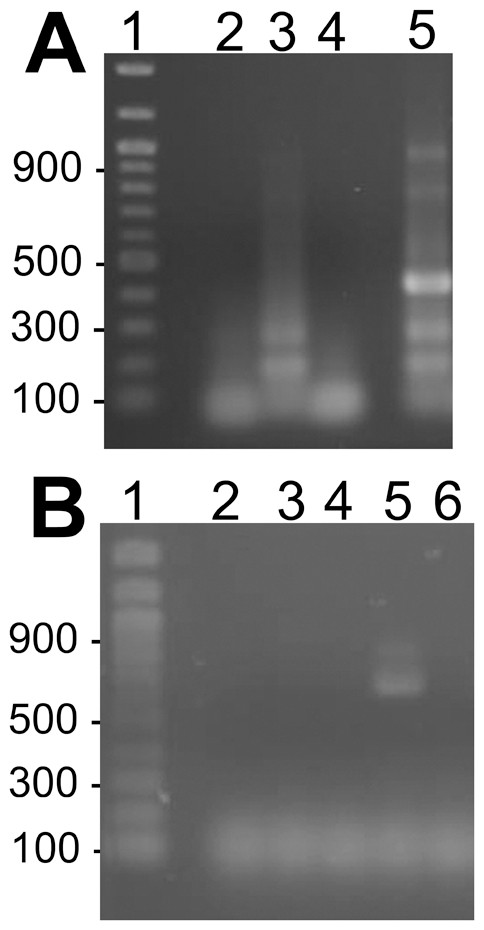
Detection of GFPuv and CAT mRNAs in Rmontanensis700 RNA extracts by RT-PCR. (**A**) RT-PCR analysis with GFPuv primers. Lane 1: 100-bp DNA marker ladder with sizes indicated at left. RT-PCR reactions were: no RNA control (lane 2); untransformed *R. montanensis* RNA (lane 3); Rmontanensis700 RNA without (lane 4) and with (lane 5) reverse transcriptase. (**B**) RT-PCR analysis with CAT primers. Lane 1 as above. RT-PCR reactions were: no RNA control (lane 2); untransformed *R. montanensis* RNA with (lane 3) and without (lane 4) reverse transcriptase; Rmontanensis700 RNA with (lane 5) and without reverse transcriptase (lane 6).

We used reverse transcriptase polymerase chain reactions (RT-PCR) and Northern blot assays to assess CAT and GFPuv mRNA expression under regulation of the *ompB* promoter. RT-PCR reactions with RNA extract from Rmontanensis700 yielded the predicted 399 bp product with nested GFPuv primers (Fig. 4A, lane 5), but control reactions without reverse transcriptase (lanes 4), without RNA (lane 2) or with untransformed *R. montanensis* RNA (lane 3) did not. Likewise, RT-PCR reactions with RNA extracted from Rmontanensis700 yielded the predicted 650 bp product with CAT primers (Fig. 4B, lane 5), but control reactions without reverse transcriptase (lane 6), without RNA (lane 2) or with untransformed *R. montanensis* RNA with (lane 3) or without reverse transcriptase (lane 4) did not. The results confirmed expression of GFPuv and CAT mRNAs in the Rmontanensis700 transformant.

**Figure 5:**
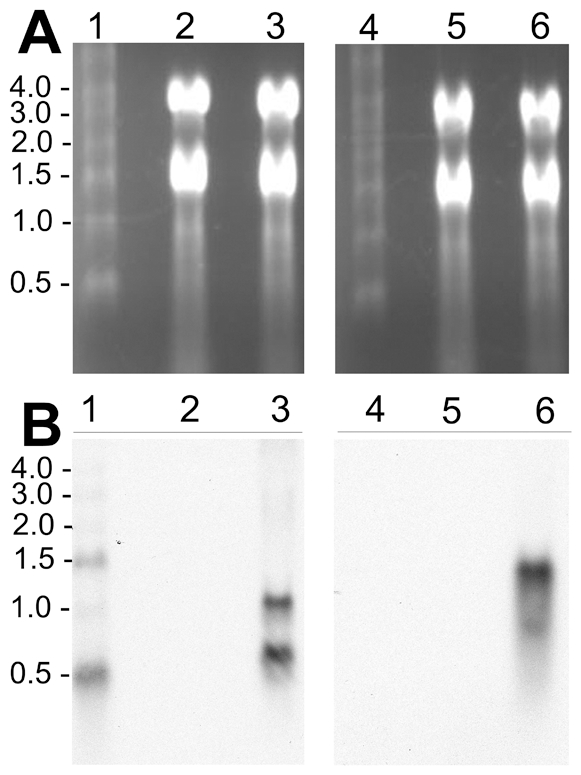
Detection of GFPuv and CAT mRNAs in Rmontanensis700 RNA extracts by Northern blotting. (**A**) Rickettsial RNA extracts electrophoresed on 1% agarose formaldehyde gels stained with EtBr. Lanes 1 and 4: 0.5 to 4.0 kb RNA marker ladder with sizes indicated at left. Lanes 2 and 5: untransformed *R. montanensis* RNA (5 µg). Lanes 3 and 6: Rmontanensis700 RNA (5 µg). (**B**) Northern blots of gels shown in panel A. Lanes 1–3 were hybridized with a GFPuv gene probe and lanes 4–6 with a CAT gene probe.

We used Northern blots to further characterize the expression profiles of the GFPuv and CAT genes in Rmontanensis700. Rickettsial RNA extracts were electrophoresed in 1% agarose formaldehyde gels and stained with EtBr. Intact ribosomal RNA migrated as bands at approximately 1.6 and 3.8 kb relative to RNA markers (Fig. 5A, lane 1) in RNA extracts of untransformed *R. montanensis* (lanes 2 and 5) and Rmontanensis700 (lanes 3 and 6). The RNAs were transferred from the gel onto nylon membranes for hybridization with GFPuv and CAT gene probes. We predicted a minimum length 920 nucleotide GFPuv mRNA transcript based on the predicted *ompB* promoter transcription start site and untranslated mRNA sequence [45] as well as transcription of 70 nucleotides of the *ompA* terminator to form mRNA hairpin structures and stop transcription [46]. Similarly, we predicted an 860 nucleotide CAT mRNA transcript. The Northern blots showed that the GFPuv and CAT probes did not hybridize to untransformed *R. montanensis* RNA (lanes 2 and 5, respectively). The GFPuv probe hybridized to unexpected 1,200 and 600 nucleotide transcripts in Rmontanensis700 RNA extracts (lane 3). Similarly, the CAT probe hybridized to unexpected 1,400 and less abundant 700 nucleotide transcripts in Rmontanensis700 RNA extracts (lane 6). The results showed that the GFPuv and CAT genes in the Rmontanensis700 transformant were both expressed under regulation of the *ompB* promoter as two transcripts that were longer and shorter than the predicted lengths.

### GFPuv Protein Expression in Transformant *R. montanensis*

**Figure 6:**
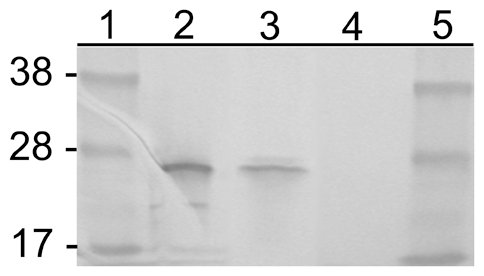
Western immunoblot of *R. montanensis* and *E. coli* protein extracts. Lanes 1 and 5: SeeBlue Plus 2 markers (Invitrogen, Carlsbad, CA) with sizes in kilodaltons indicated at left. Lane 2: *E. coli* transformed with pMOD700 plasmid. Lane 3: Rmontanensis700 transformed with the pMOD700 transposon. Lane 4: Untransformed *R. montanensis*. Ten µg protein were loaded per lane and the blot was probed with anti-GFP antibody.

Because the Rmontanensis700 transformant produced unexpected GFPuv mRNA transcripts and it was less fluorescent than the Rmona658 transformant that produced the expected GFPuv mRNA transcript [39], we used Western immunoblots to study expression of the GFPuv protein. No protein in untransformed *R. montanensis* control extracts (Fig. 6, lane 4) was recognized by a polyclonal anti-GFP antibody but extracts of Rmontanensis700 (lane 3) and *E. coli* transformed with pMOD700 plasmid (lane2) contained proteins that recognized by the antibody as major bands that co-migrated at the predicted GFPuv molecular mass of 26 kDa relative to marker protein bands (lanes 1 and 5). The results indicated that Rmontanensis700 and *E. coli* expressed a predominant GFPuv protein species of the predicted molecular mass despite the expression of both larger and smaller than expected GFPuv mRNA transcripts in Rmontanensis700 (Fig. 5B, lane 3).

## Discussion

Transposon-based transformation of *R. montanensis* follows that of *R. prowazekii* [37], [38], [41] and *R. monacensis* [39], [30], albeit without the high efficiency typical of plasmid transformation of bacteria. The long-standing barriers to transformation of rickettsiae [33] have been overcome, allowing evaluation of transcriptional regulatory elements, selectable markers and reporter genes for use in further development of rickettsial transformation technologies. The currently available transformant rickettsiae expressing fluorescent reporter genes are of immediate practical value by allowing visualization of living rickettsiae in unfixed host tissues as in the case of GFP-fluorescent *R. monacensis* in *Ixodes scapularis* [47]. The Rmontanensis700 transformant can be used to enhance the *R. montanensis* in *D. variabilis* model system [30], [31], [32].

Earlier efforts to transform rickettsiae relied on recombination of selectable marker or GFP reporter genes unaccompanied by a promoter into chromosomal loci under regulation of an active rickettsial promoter [48], [49], [50], or recombination of a reporter gene under regulation of a cloned *E. coli* promoter [51]. The transformant rickettsiae had low level and/or unstable expression of the foreign gene. Although the present transposon strategy using cloned rickettsial promoters to regulate gene expression has been much more successful, only four promoters have been used to date. The *R. prowazekii rpsL*, the *R. rickettsii ompA* and the *Borrelia flg* promoters have been used to express a rifampin resistance factor, GFPuv and the Himar1 transposase, respectively, in *R. prowazekii*, but without evaluation of mRNA transcription or protein expression [37], [41]. The *ompA* promoter was used to express the GFPuv and CAT genes in a *R. monacensis* transposon-mediated transformant, Rmona658 [39]. Northern blots showed that single mRNAs of predicted length were expressed from both genes and the expected GFPuv protein was observed on Western immunoblots. A second transformant (Rmona601) correctly expressed CAT mRNA under regulation of the *ompA* promoter but the DsRed2 gene, under regulation of the *ompB* promoter, was expressed as full-length and half-length mRNAs that were not translated, probably due to extreme GC-rich codon bias versus the AT-rich codon bias of rickettsiae [39]. In this report, we have shown that the *ompB* promoter-driven CAT and GFPuv genes in Rmontanensis700 were both expressed as two mRNA species that were larger and smaller than predicted. The larger CAT mRNA transcript was much more abundant than the smaller transcript but the larger and smaller GFPuv transcripts were of approximately equivalent abundance (Fig. 5). Western immunoblots nevertheless showed that Rmontanensis700 protein extracts contained a GFPuv protein of the expected mass that co-migrated with GFPuv protein expressed by *E. coli* transformed with the pMOD700 transposon [Fig. 6]. The low GFPuv fluorescence intensity of Rmontanensis700 rickettsiae, as compared to that of Rmona658 rickettsiae, was therefore not due to expression of a compromised GFPuv protein. While we did not compare CAT activity in the transformant rickettsiae, the 3-fold greater period of time required to obtain the Rmontanensis700 line under a 2-fold lower concentration of CHL than that required to obtain the Rmona658 line in the same host cell line [39] suggested a relatively lower level of CAT activity in Rmontanensis700.

With the exception of the difference in promoters, the pMOD658 and pMOD700 transposons used to transform *R. monacensis* and *R. montanensis*, respectively, were essentially identical. In both transposons, the GFPuv and CAT expression cassettes were in tandem head to tail orientation, each with an independent *ompA* transcription terminator. In the Rmona601 transformant, the predicted CAT mRNA was expressed from pMOD601 in which the ompACAT and ompBDsRed2 expression cassettes were in opposed head to tail orientation with a single shared *ompA* terminator [39]. The *ompB* promoters in Rmona601 and Rmontanensis700 drove expression of aberrant DsRed2, GFPuv and CAT mRNAs regardless of the orientations of the expression cassettes or the presence of independent or single shared transcription terminators. In contrast, the *ompA* promoter drove expression of the predicted GFPuv and CAT mRNAs from both tandem or opposed head-to-tail expression cassettes within the same transposon.

The *R. rickettsii ompA* and *ompB* promoter transcription start sites and their relative activities were originally characterized on the basis of primer extension and Northern blot analyses of RNA extracts from *E. coli* transformed with a promoter assay vector containing a CAT reporter gene [45]. Steady state CAT mRNA levels and CAT protein levels in *E. coli* containing the *ompB* promoter version of the vector were approximately 2-fold and 28-fold higher, respectively than those of *E. coli* containing the *ompA* promoter version of the vector. The results implied significant differences in mRNA stability and/or efficiency of translation, possibly influenced by sequences present in the approximately 120 and 20 nucleotide 5′ untranslated regions of the *ompB* and *ompA* promoter-regulated mRNAs, respectively. In *R. rickettsii*, the relative levels of OmpB protein were approximately 9-fold higher than those of OmpA and the authors stated that preliminary data indicated that *ompB* mRNA half-life was several-fold longer than that of *ompA* mRNA. Those results suggested to us that the *R. rickettsii ompB* promoter would drive higher gene expression than the *ompA* promoter in transformant rickettsiae. Our results have indicated the opposite.

We have demonstrated the apparent practical superiority of the *R. rickettsii ompA* promoter over the *ompB* promoter as a regulator of foreign gene expression in two species of transformant rickettsiae. However, it is not clear whether that was due to species-specific differences in promoter activity and transcript fidelity without the influence of transposon integration site effects. Accurate evaluation of promoters for expression of foreign genes in transformant rickettsiae awaits availability of a rickettsial transformation vector that allows direct comparison of gene expression constructs differing only in promoters and free of possible chromosome integration effects. The recently discovered plasmids of the *Rickettsia* [40], [52], [53] may provide a basis for development of such a vector.
